# Transcriptome Analysis of Cell Wall and NAC Domain Transcription Factor Genes during *Elaeis guineensis* Fruit Ripening: Evidence for Widespread Conservation within Monocot and Eudicot Lineages

**DOI:** 10.3389/fpls.2017.00603

**Published:** 2017-04-25

**Authors:** Timothy J. Tranbarger, Kim Fooyontphanich, Peerapat Roongsattham, Maxime Pizot, Myriam Collin, Chatchawan Jantasuriyarat, Potjamarn Suraninpong, Somvong Tragoonrung, Stéphane Dussert, Jean-Luc Verdeil, Fabienne Morcillo

**Affiliations:** ^1^Institut de Recherche pour le Développement, IRD, UMR DIADEMontpellier, France; ^2^Department of Genetics, Kasetsart UniversityBangkok, Thailand; ^3^Department of Plant Science, Institute of Agricultural Technology, Walailak UniversityNakhon Si Thammarat, Thailand; ^4^Genome Institute, National Center for Genetic Engineering and BiotechnologyPathumthani, Thailand; ^5^Centre de Coopération Internationale en Recherche Agronomique pour le Développement, UMR AGAPMontpellier, France; ^6^Centre de Coopération Internationale en Recherche Agronomique pour le Développement, UMR DIADEMontpellier, France

**Keywords:** ripening, cell wall, NAC domain, oil palm, monocotyledon, mesocarp, ethylene

## Abstract

The oil palm (*Elaeis guineensis*), a monocotyledonous species in the family Arecaceae, has an extraordinarily oil rich fleshy mesocarp, and presents an original model to examine the ripening processes and regulation in this particular monocot fruit. Histochemical analysis and cell parameter measurements revealed cell wall and middle lamella expansion and degradation during ripening and in response to ethylene. Cell wall related transcript profiles suggest a transition from synthesis to degradation is under transcriptional control during ripening, in particular a switch from cellulose, hemicellulose, and pectin synthesis to hydrolysis and degradation. The data provide evidence for the transcriptional activation of expansin, polygalacturonase, mannosidase, beta-galactosidase, and xyloglucan endotransglucosylase/hydrolase proteins in the ripening oil palm mesocarp, suggesting widespread conservation of these activities during ripening for monocotyledonous and eudicotyledonous fruit types. Profiling of the most abundant oil palm polygalacturonase (*EgPG4*) and 1-aminocyclopropane-1-carboxylic acid oxidase (*ACO*) transcripts during development and in response to ethylene demonstrated both are sensitive markers of ethylene production and inducible gene expression during mesocarp ripening, and provide evidence for a conserved regulatory module between ethylene and cell wall pectin degradation. A comprehensive analysis of NAC transcription factors confirmed at least 10 transcripts from diverse NAC domain clades are expressed in the mesocarp during ripening, four of which are induced by ethylene treatment, with the two most inducible (*EgNAC6* and *EgNAC7*) phylogenetically similar to the tomato NAC-NOR master-ripening regulator. Overall, the results provide evidence that despite the phylogenetic distance of the oil palm within the family Arecaceae from the most extensively studied monocot banana fruit, it appears ripening of divergent monocot and eudicot fruit lineages are regulated by evolutionarily conserved molecular physiological processes.

## Introduction

Fruit ripening is a biological character unique to flowering plants, not only of central importance to seed dispersal and the reproductive success of plants, but also essential for both human and animal diets. The color, metabolic, and textural transitions that occur in the ripening fleshy fruit tissues have economic and nutritional consequences for humans. Ethylene is known to play a central role in the ripening of climacteric fruit in which there is a shift from a basal non-catalytic (system 1) to an autocatalytic (system 2) increase in ethylene production (Lelievre et al., 1997; Klee and Giovannoni, 2011; Liu et al., 2015). Studies with the climacteric fruit model tomato indicate a central role for ethylene in the transcriptional coordination of metabolic processes that occur during ripening (Alba et al., 2005; Osorio et al., 2011). The role of ethylene is conserved across many eudicot species and is likely to play similar roles in other climacteric fleshy fruits such apple, peach, pear, mango, and banana (Prasanna et al., 2007; Bapat et al., 2010; Seymour et al., 2013a,b). Furthermore, recent studies indicate that ethylene signal transduction is also important for the transcriptional regulation or ripening process in non-climacteric fruit that lack the large climacteric increase in ethylene, such as pepper, grape, and strawberry (Trainotti et al., 2005; Chervin et al., 2008; Bapat et al., 2010; Osorio et al., 2012). While ethylene may be viewed as a common regulator for fruit ripening in general, how the ripening related regulatory networks have evolved and adapted during plant evolution is unknown. Indeed, most of the research on fruit ripening focuses on the important cultivated eudicotyledonous species, in particular tomato. However, research with the monocotyledonous banana and the basal angiosperm *Persea americana* (avocado) support the notion that the role of ethylene has been conserved through plant evolution, but given the large phylogenetic distances of these species, diversification can be expected to be discovered (Chanderbali et al., 2008, 2009; Elitzur et al., 2010, 2016; Jourda et al., 2014).

One of the most important processes regulated by ethylene during fruit ripening are the textural changes that occur due to cell wall disassembly, indeed, transgenic tomato with modified ethylene biosynthesis or perception have provided evidence that ethylene regulated cell wall metabolism is central to climacteric fruit ripening (Bennett and Labavitch, 2008). However, while extensive work has been done with the ripening tomato as a eudicot model, our understanding of how the cell wall is modified for softening to occur is still incomplete. In general, it appears that textural changes during ripening are controlled simultaneously by many genes, and may be species and/or fruit type dependent (Bennett and Labavitch, 2008; Seymour et al., 2013a).

NAC-NOR is an example of a master regulator that controls ethylene production and downstream changes in the cell wall of the ripening tomato (Giovannoni, 2004; Seymour et al., 2013a). The *non-ripening* (*nor*) tomato mutation affects a NAC domain transcription factor (TF) and results in fruit that do not ripening normally, similar to both *ripening-inhibitor* (*rin*) and *never-ripe* (*nr*) (Giovannoni, 2004; Osorio et al., 2011). All these mutants share a strong down regulation of the tomato fruit polygalacturonase (TFPG) transcript (*PG2*), indicating that the transcriptional activation of the tomato *PG2* gene is an important control point regulating the expression of *PG2* during ripening (Dellapenna et al., 1986, 1987, 1989). While PG can hydrolyze the backbone of pectin homogalacturonan polymers, the down-regulation or knockout of the TFPG transcript results in a decrease in pectin depolymerization, but no change in fruit softening, and it is generally thought that a large number of downstream cell wall related target genes are activated transcriptionally during ripening and are necessary for softening (Sheehy et al., 1988; Smith et al., 1988, 1990; Cooley and Yoder, 1998; Brummell and Harpster, 2001; Brummell, 2006; Seymour et al., 2013a). NAC domain proteins have emerged as important transcriptional regulators of tomato fruit ripening. For example, the silencing of two NAC domain TFs *SNAC4* and *SNAC9* results in decreased expression of the tomato ethylene biosynthesis genes *LeACS2, LeACS4* (encoding 1-amino-cyclopropane-1-carboxylic acid or ACC synthases), and *LeACO1* (tomato ACC oxidase), which consequently inhibition the ripening process (Kou et al., 2016). Indeed, the SNAC TFs bind to the promoter of these ethylene biosynthesis genes *in vitro*, while the silencing of *LeACS4, LeACO1*, and *LeERF2* reduced the expression of *SNAC4* and *SNAC9*, suggesting a possible feedback system between these SNAC TFs and ethylene biosynthesis gene transcription. In contrast, less is known about monocot fruit ripening. Recent studies found NAC domain transcripts were differentially expressed in the ripening oil palm mesocarp, suggesting ethylene-regulated expression, while a study with banana identified at least 6 NAC domain TFs expressed during banana ripening, two of which are induced by ethylene (Tranbarger et al., 2011; Shan et al., 2012). Together these results suggest that NAC domain TFs may have conserved central roles during ethylene induced fruit ripening of both eudicots and monocots.

The monocot–eudicot divergence occurred 130–135 million years ago (D'hont et al., 2012; Magallón et al., 2015). The best-studied monocot fruit is banana in which ethylene plays a conserved role during ripening (Elitzur et al., 2010, 2016; Jourda et al., 2014). Arecaceae (the monocot palm family) displays a very large amount of fruit diversity and is separated from its closest monocot lineage by approximately 98 million years (Magallón et al., 2015). Ethylene appears to be involved in fruit ripening of both oil palm (*Elaeis guineensis*) and date palm (*Phoenix dactylifera* L), while oil palm has twice the number of ripening and abscission genes and more *ACO* genes than date palm (Abbas and Ibrahim, 1996; Tranbarger et al., 2011; Singh et al., 2013; Nualwijit and Lerslerwong, 2014). Recent studies by our group identified a polygalacturonase (*EgPG4*) highly induced by ethylene in oil palm fruit abscission zone cells and associated with the cell separation and fruit abscission (Roongsattham et al., 2012, 2016). Whereas, *EgPG4* is not expressed or induced in the adjacent pedicel tissue, it is also induced by ethylene and highly expressed in the mesocarp cells (Roongsattham et al., 2012). In addition, quantitative changes in the mesocarp cell walls occur during development and ripening, including thickness increases in the primary cell wall and middle lamella, while ethylene treatment of ripe fruit results in a decrease in middle lamella thickness (Roongsattham et al., 2016).

The majority of our knowledge about ethylene function during fruit ripening in higher plants comes from studies with eudicot model plants, in particular with the tomato fruit, and to a lesser extent with the monocot model fruit banana. To what extent this knowledge extends to non-model and phylogenetically distant species is not fully known. The current study focuses on the ethylene and cell wall related processes that take place during the ripening of the oil palm fruit mescarp in comparison with what is known from the main eudicot and monocot fruit models, banana, and tomato.

## Materials and methods

### Plant material, treatments, and RNA extraction

Oil palm fruits for the mesocarp transcriptome were harvested at Pobè CRA-PP Station (INRAB) Benin, from a *dura* parent of Deli Dabou origin, within the same self-progeny of a single palm as described previously (Tranbarger et al., 2011). Oil palm spikelets with ripe fruit 180 days after pollination (DAP), at which time fruit are typically harvested and transported for oil extraction, were sampled from a tenera clone (clone C) at the Krabi Golden Tenera plantation, Thailand as previously described (Roongsattham et al., 2012). Spikelets were treated for 3, 6, and 9 h with 10 μl l^−1^ ethylene, a quantity of ethylene that was previously found to induce changes to the cell walls of the fruit abscission zone cells that results in 25% fruit abscission after 9 h (Roongsattham et al., 2012, 2016). For RNA extractions, mesocarp fruit tissue samples were collected and frozen immediately in liquid nitrogen. Total RNA from mesocarp was extracted as previously described (Morcillo et al., 2006). Total RNA (1 μg) was used to synthesize cDNA using the first-strand cDNA synthesis kit (ImProm-II™ Reverse Transcription System, Promega).

### Quantitative real-time RT-PCR

qPCR was conducted as previously described (Roongsattham et al., 2012). The analysis was conducted on a LightCycler 480 (Roche) in 96-well plates in a volume of 10 μl containing 2 μl of cDNA diluted 1/100, 1.5 μl of primer forward (2 μM), 1.5 μl of reverse primer (2 μM), and 5 μl SYBR® Green Mastermix (Roche). Primers used for *EgPG4* and *ACO* amplification were previously published (Fooyontphanich et al., 2016). PCR was initiated by denaturation at 95°C for 10 min, followed by 45 cycles of 95°C for 15 s, 60°C for 15 s, and a final extension at 70°C for 1 min. All expression was normalized to the *EgEf* α1 (accession number: AY550990) mRNA from *E. guineensis*, and mRNA abundance for each experiment was calculated relative to the sample with the lowest amount of transcript present determined with the formula as described previously (Pfaffl, 2001). No change of *EgEf*α*1* transcript accumulation was found in the fruit tissues treated or not treated with ethylene. Control using RNA matrices were also conducted to validate the absence of DNA in each sample. Each time point was replicated three times from two independent biological samples, and all amplified cDNA fragments were sequenced by Beckman-Cogenics (https://www.genewiz.com) to check the specificity of the amplified products. Gene abundance is expressed as mean and standard error bars are calculated from the technical replicates of one of the biological repetitions.

### Mesocarp transcriptome data mining

Transcriptome data of the developing mesocarp previously clustered (clusters A, B, C, and D) was searched for transcripts with expression profiles that either increase or decrease during the burst of ethylene production observed during oil palm fruit ripening between 100 and 160 DAP (Supplementary Table 1; Tranbarger et al., 2011). The BLAST2GO and InterProScan web services with BLASTX using an E-value cutoff of 1e-5 were used to annotate the gene sets (Altschul et al., 1990; Zdobnov and Apweiler, 2001; Götz et al., 2008). Cell wall sequences were identified by searching the GO annotated sequences for InterPro accessions and key words related to cell wall processes, and by searching (TBLASTX) the 454 sequence database with known candidates related to cell wall biosynthesis and degradation.

### RNA-seq data processing

Nine RNAseq datasets generated by Orion Genomics (http://www.oriongenomics.com/index.php/en/) for selected *E. guineensis* tissues were downloaded from NCBI databases: 17 cm long leaf (SRX278048), spear leaf (SRX278049), 2.5 cm (SRX278052), and 20 cm long female inflorescence (SRX278053), pollen (SRX278051), root (SRX278062), shoot apex (SRX278055), and kernel at 10 and 15 weeks after anthesis (SRX278021 and SRX278018, respectively). Reads from these datasets in addition to RNAseq data (100-nt Illumina reads) for the *E. guineensis* ripening mesocarp (Guerin et al., 2016) and embryonic cell suspensions (kindly provided by Thierry Beulé) were then mapped on the *E. guineensis* CDS reference (NCBI website, GCF_000442705.1_EG5_rna.fna; January 2015) and the RPKM (number of reads per kilobase and million reads) for each locus were compared.

### Transcription factor prediction

The NAC domain gene family of *E. guineensis* was retrieved from the Plant Transcription Factor Database (PlantTFDB, http://planttfdb.cbi.pku.edu.cn/prediction.php) 4.0 using the *E. guineensis* CDS reference (NCBI website, GCF_000442705.1_EG5_rna.fna; January 2015; Jin et al., 2015, 2016). From a total of 44,360 oil palm putative coding sequences (CDS), 175 NAC domain-containing sequences were identified in the oil palm reference CDS, which corresponded to a total of 124 non-redundant loci. The same result was obtained using the iTAK database (Zheng et al., 2016; http://bioinfo.bti.cornell.edu/cgi-bin/itak/online_itak.cgi).

### Phylogenetic analysis

Amino acid alignments were performed with ClustalW (Larkin et al., 2007) using default settings (http://www.ebi.ac.uk/). The amino acid sequences containing the conserved NAC subdomains (A–E) were used for the phylogenetic analysis (Ooka et al., 2003) using the default settings without the G-blocks step (Dereeper et al., 2008). Branch support values are based on the aLRT statistical test (Anisimova and Gascuel, 2006).

### Histological analysis and cell measurements

Mesocarp fruit samples were collected from ethylene treated fruit and fixed in 0.2 M phosphate buffer containing 2% (w/v) paraformaldehyde, 1% (w/v) caffeine, and 0.5% (v/v) glutaraldehyde or a minimum of 2 days at 4°C as previously described (Buffard-Morel et al., 1992). Serial dehydration with ethanol from 30 to 100%, then 100% butanol/100% ethanol (v/v), and finally 100% butanol was performed for each sample and followed by impregnation and embedding in Technovit 7100 resin (Heraeus Kulzer). Semi-thin sections of 3 μm were cut using a microtome. Each section was stained with toluidine blue or ruthenium red. Microphotographs were taken with a Leica camera (DFC 300 FX) on a Leica (LEITZ DMRB) light microscope (x20/0.5; x40/0.7; and x100/1.3). Cell parameter measurements were performed for cell width, cell wall width, and middle lamella as previously reported (Roongsattham et al., 2016).

## Results

### *EgACO* and polygalacturonase *EgPG4* transcript profiling during mesocarp development and in response to ethylene treatment of ripe fruit

**Figure 1:**
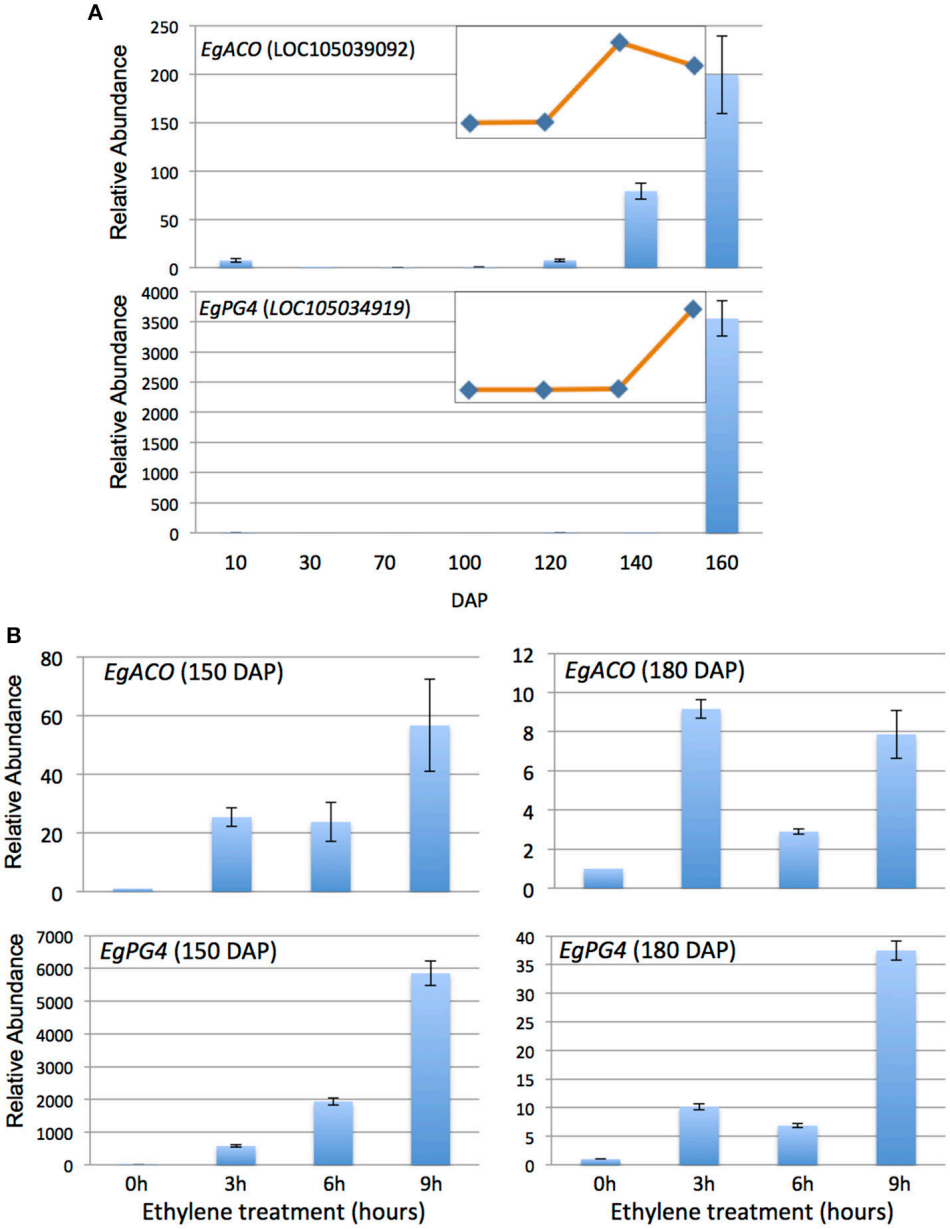
*****EgACO*** and ***EgPG4*** expression in the oil palm mesocarp increase during fruit ripening and are induced by external ethylene treatments at two different ripening stages**. qPCR analysis of *EgACO* and *EgPG4* transcript abundance in the mesocarp during development **(A)** and in response to exogenous ethylene (10 μL l^−1^) treatments **(B)**. Inserts are RNA-Seq based RPKM profiles of the mesocarp at 100, 120, 140, and 160 DAP.

To provide molecular indicators of ethylene production in the mesocarp, we examined the quantitative expression profiles of the most highly abundant *ACO* previously observed in the oil palm mesocarp (CL1Contig999, *EgACO*), in addition to the most highly expressed polygalacturonase (CL1Contig5616, *EgPG4*) in the mesocarp induced by ethylene (Tranbarger et al., 2011; Roongsattham et al., 2012; Fooyontphanich et al., 2016) (Supplementary Tables 1, 2). The *EgACO* nucleotide sequence aligns most significantly (E-value 0) to LOC105039092 (Chromosome 2) annotated as an *ACO3* in the oil palm genome (Supplementary Table 2). During mesocarp development, the *EgACO* transcript is expressed in the pollinated flower at the onset of fruit development (10 DAP) and is undetectable or barely detectable until 120 DAP, and then increases 79 and 199 fold at 140 and 160 DAP respectively (Figure 1A). Illumina sequencing confirmed that the *EgACO3* transcript increased between 120 and 160 DAP (Figure 1A, Supplementary Table 2). The pattern of *EgACO* expression correlates to the increase in ethylene observed between 120 and 160 DAP, which corresponds to the transition from system 1 to system 2 ethylene production (Tranbarger et al., 2011). To determine whether the *EgACO* transcript could be regulated by ethylene, the amount of *EgACO* transcript was quantified in the mesocarp of ripe fruit (150 and 180 DAP) treated with ethylene for 3–9 h (Figure 1B). After 3 h of ethylene treatment, in 150 DAP fruit the *EgACO* transcript increased 25 fold, while in the 180 DAP fruit, the transcript increased 9 fold. A further increase was observed at 9 h while at 6 h the *EgACO* transcript amounts remained stable (150 DAP) or decreased (180 DAP). In contrast to *EgACO*, the *EgPG4* transcript is undetectable or present at very low amounts from 10 to 140 DAP, then increases dramatically over 3,000 fold at 160 DAP, which corresponds to the autocatalytic system 2 ethylene production (Figure 1B). *EgPG4* is annotated at LOC105034919 (Chromosome 2, Supplementary Table 2). Illumina sequencing confirmed an increase of *EgPG4* transcript increased between 140 and 160 DAP (Figure 1A, Supplementary Table 2). The *EgPG4* transcript increased in both 150 and 180 DAP fruit after 3h of ethylene treatment, but the magnitude was much higher in 150 DAP (500 fold) than in the older 180 DAP fruit (10 fold). The *EgPG4* transcript continued to increase after 6 and 9 h of ethylene treatment and eventually reached over 5,000 fold by 9h in 150 DAP fruit. While both the *EgACO* and *EgPG4* transcripts increase rapidly (3 h) in response to ethylene treatment, the induction was less in 180 DAP fruit for both genes, while the magnitude of induction was consistently higher for *EgPG4*. Furthermore, *EgPG4* expression is limited to the ripest fruit in contrast to *EgACO*, which is also expressed 10 days after the flower is pollinated.

### Histological analysis of the mesocarp in response to ethylene in ripening fruit

**Figure 2:**
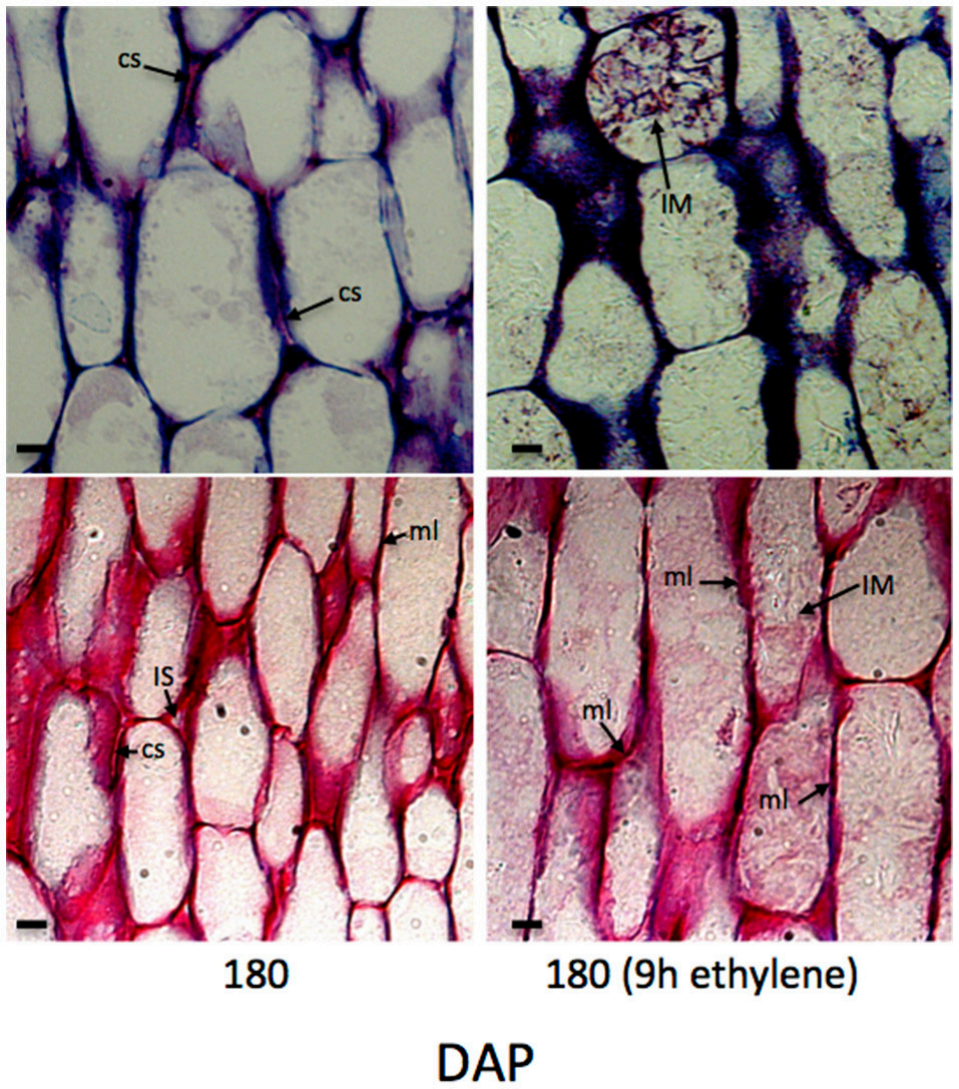
**Mesocarp cell characteristics change during development and after 9 h ethylene treatment**. A longitudinal sections of mesocarp cells toward the base of the fruit stained with toluidine blue (upper panels) and ruthenium red (lower panels). cs, cell separation; dc, divided cell; IM, intercellular material; IS, intercellular space; ml, middle lamella; Scale bar, 10 μm.

Histological analysis of abscission zone and mesocarp cell walls revealed changes in integrity during development and in response to ethylene treatments (Roongsattham et al., 2016). While the previous article focused on cellular changes in abscission zone cells, here we focus on changes that occur in the mesocarp cells during ripening and in response to ethylene (Supplementary Figures 1, 2). To examine the cellular processes in the mesocarp, longitudinal tissue sections were analyzed from samples of mesocarp at key stages of development and ripening, and in response to ethylene. At 30 DAP, anticlinal cell divisions have taken place, cells have not fully elongated, and the formation of intercellular spaces is observed (Roongsattham et al., 2016; Supplementary Figure 1). By 120 DAP, cells have elongated and a significant increase in the cell wall width is observed, with the average cell wall width more than 1 μm while the middle lamella is approximately 0.6 μm wide and the presence of intercellular spaces is also observed at 120 DAP. At 180 DAP, the cell wall width increased, and cell separation between adjacent cells is apparent. By 180 DAP a 2-fold increase in cell wall width is observed, and the middle lamella width also increases significantly to ~0.9 μm. Based on these measurements, the cell wall accounts for approximately 4, 7, and 15% of the total cell width at 30, 120, and 180 DAP respectively. After 9 h of ethylene treatment of 180 DAP fruit, the toluidine blue staining increases between adjacent cells, while ruthenium red stained sections reveal non-continuous dark strands that corresponds to an apparent breakdown of the middle lamella that is measurable after 9 h of ethylene treatment (Figure 2). Finally, in ethylene treated cells, intercellular material accumulates based on both toluidine blue and ruthenium red staining (Figure 2). Based on these data, a significant amount of cell wall and the middle lamella expansion occurs between 30 to 180 DAP, while the integrity of the middle lamella in the mesocarp cells is most affected by ethylene treatment of 180 DAP fruit (Figure 2).

### Cell wall associated transcriptional activity during mesocarp development and ripening

**Figure 3:**
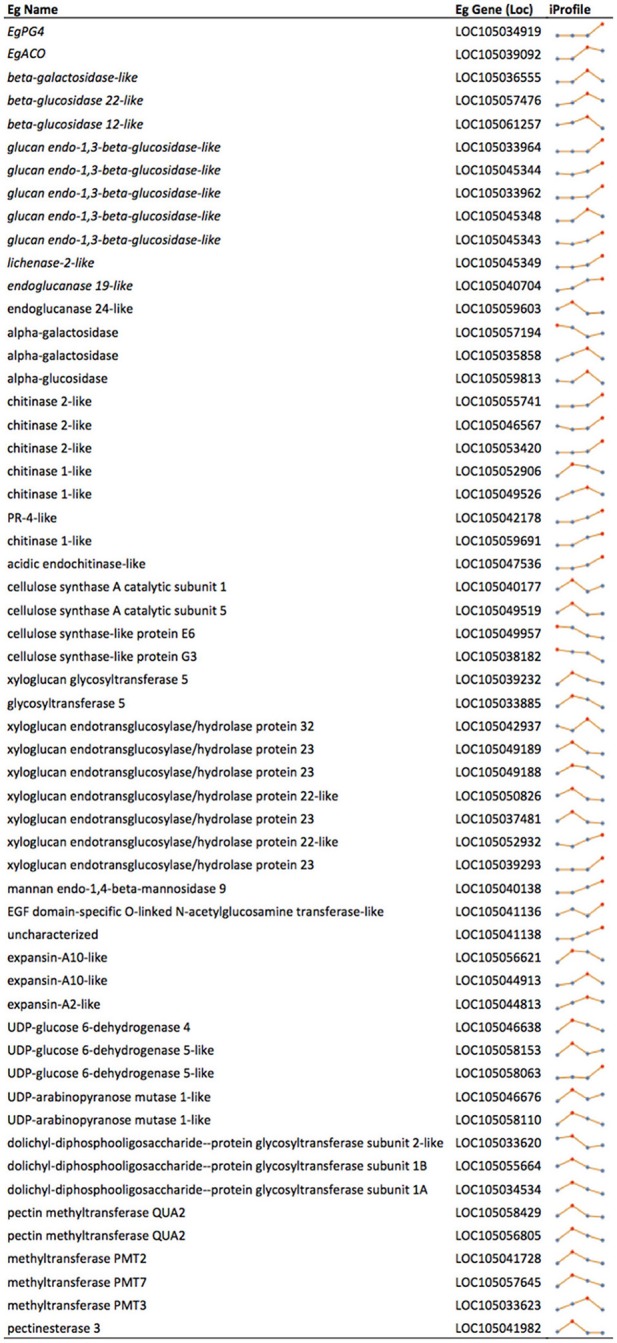
**Cell wall related transcripts with expression profiles in the mesocarp correlated to ripening stages concomitant with the increase in ethylene evolution at 140 and 160 DAP**. *EgACO* and *EgPG4* are included for a comparison as known ethylene inducible transcripts. Profiles are based on RNA-Seq Illumina (iProfile) sequencing at each developmental time point and expressed as RKPM (sum of all possible transcripts, XMs, for each gene locus) A, 100 DAP; B, 120 DAP; C; 140 DAP; C, 160 DAP; Eg, *E. guineensis*. Red dot in profiles indicates maximum read peak.

**Figure 4:**
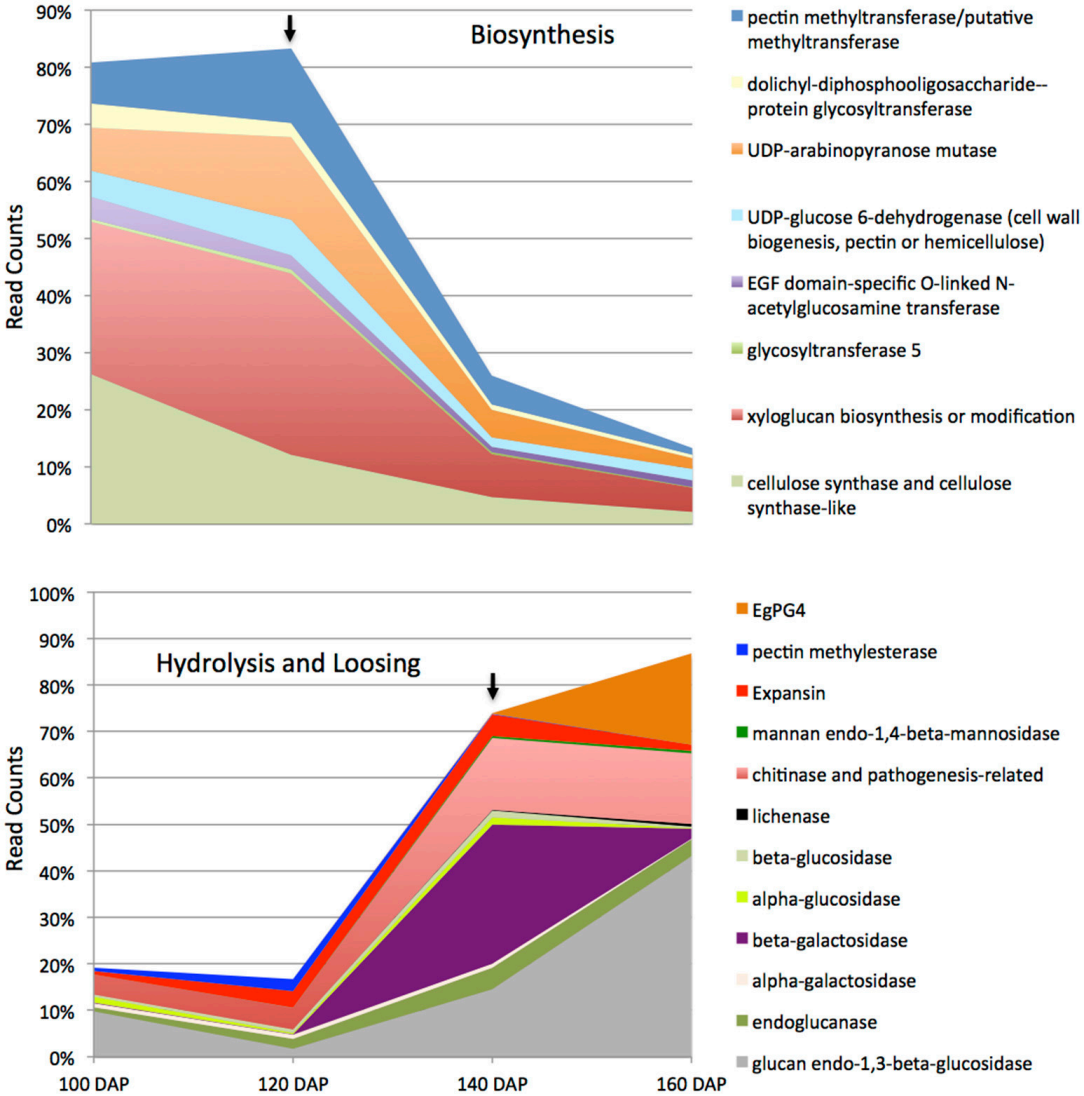
**Global cell wall related transcript abundance during mesocarp development and ripening**. The transcripts and their encoded products can be grouped into two categories including biosynthesis (upper panel) and hydrolysis (lower panel) related functions. The upper black arrow indicates the stage at which the transition from ethylene system 1 to system 2 occurs while the lower arrow indicates the stage at which the ethylene burst occurs (Tranbarger et al., 2011). See Supplementary Tables 1, 2 for details.

Based on the histological analysis, an increase in cell wall width occurs during mesocarp development and ripening, while exogenous ethylene treatment results in a loss of middle lamella integrity (Figure 2; Roongsattham et al., 2016). To provide insight into the transcriptional basis for these processes, proteins associated with cell wall assembly, metabolism, and modification were searched for within the differentially abundant transcripts previously identified in the mesocarp transcriptome at 100, 120, 140, and 160 DAP (Supplementary Table 1; Tranbarger et al., 2011). A total of 75 transcripts for cell wall related activities were found to be differentially expressed in the mesocarp during ripening, 63% of which have expression peaks at 140 and 160 DAP (including *EgPG4*) concomitant with the ethylene burst as measured previously (Tranbarger et al., 2011). Illumina sequencing at 100, 120, 140, and 160 DAP mesocarp validated the expression 54 of these genes annotated in the oil palm genome with roles in cell wall component biosynthesis, assembly, modification and hydrolysis during fruit ripening (Figure 3; Supplementary Table 2; Guerin et al., 2016). A global comparison of each transcript type expressed as a percentage of all cell wall transcripts gives an estimation of the transcriptional contribution of each cell wall activity during these stages of fruit ripening (Figure 4; Supplementary Table 3). Globally, the most abundant transcripts encode sequences similar to glucan endo-1,3-beta-glucosidase, with 25% of the total read counts from the 4 stages of development, and a peak of 43% at 160 DAP. Chitinase and pathogenesis-related proteins accounted for 13% of the total, with 16% and 15% at 140 DAP and 160 DAP respectively, while xyloglucan biosynthesis or modification proteins accounted for 12% of the total with 32% at 120 DAP (Figure 4, Supplementary Table 3). Xyloglucan biosynthesis or modification proteins were also highly abundant (27%) along with cellulose synthase and cellulose synthase-like transcripts (26%) at 100 DAP. Transcripts for cellulose synthase and cellulose-synthase like proteins decreased progressively during the later stages to 12% (120 DAP), 5% (140 DAP), and 2% (160 DAP). Transcripts for uridine diphosphate (UDP) glucose 6-dehydrogenase (9%), involved in both pectin and hemicellulose biogenesis, were more abundant at 120 DAP, but also present throughout the developmental stages examined (Klinghammer and Tenhaken, 2007). Pectin related transcripts included pectin methyltransferases (PMTs, 13% 120 DAP), a pectin methylesterase (PME, 3% 120 DAP) and the transcript *EgPG4*, which represents 20% of the total cell wall related transcripts at 160 DAP (Figure 3). Overall, there is a progressive change from cell wall biosynthesis to hydrolysis during the transition from ethylene system 1 to 2 (between 120 and 140 DAP) that includes transcripts encoding glucan endo-1,3-beta-glucosidase, endoglucanase, beta- and alpha-galactosidase, chitinase and pathogenesis-related, mannan endo-1,4-beta-mannosidase and expansin proteins. Between 140 and 160 DAP, the transcripts that increased the most were *EgPG4* (210 fold) and glucan endo-1,3-beta-glucosidase-like (LOC105033964, ~350 fold; Supplementary Table 3). In addition, one transcript similar to a xyloglucan endotransglucosylase/hydrolase protein 23 (LOC105039293) transcript was expressed at relatively high amounts exclusively at 160 DAP. The profile similarity of *EgPG4* with other transcripts suggest certain cell wall activities may be coordinated by ethylene during mesocarp ripening (Figure 4, Supplementary Tables 2, 3).

### Analysis of NAC transcription factor transcripts expressed in the mesocarp and in response to ethylene

**Figure 5:**
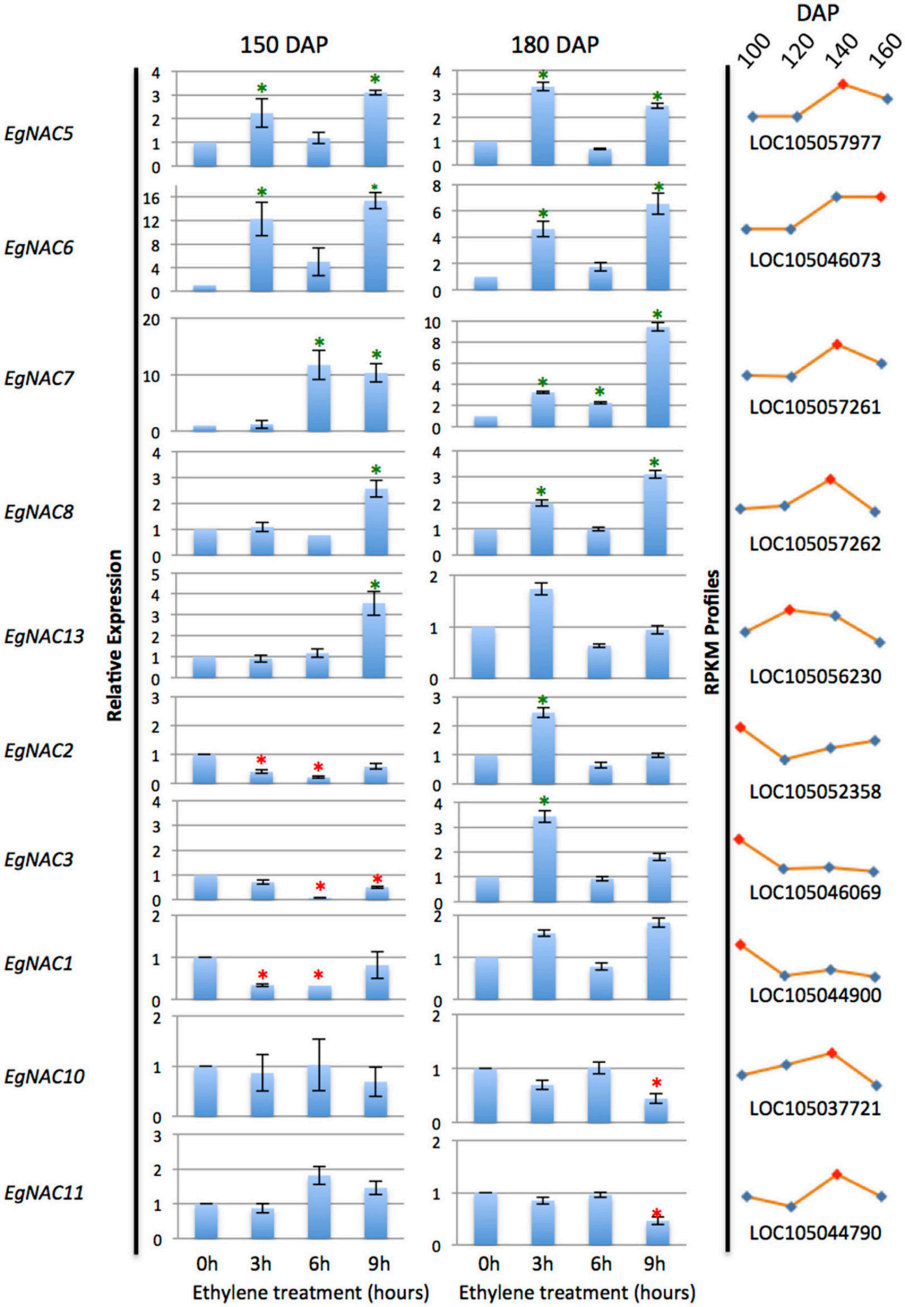
**qPCR analysis of NAC transcripts expressed in the mesocarp and induction in response to ethylene treatments**. Asterisks indicate significant difference and increase or decrease in relative abundance by at least 2 fold (unpaired student's *t*-test *P* > 0.05) compared to the control at 0 h. Profile curves (right panels) are the corresponding *E. guineensis* gene locus expression during ripening at 100, 120, 140, and 160 DAP from RNA-Seq Illumina data.

NAC domain containing TFs were found previously to be one of the most highly represented TFs expressed in the mesocarp (Tranbarger et al., 2011). To respond to the question whether any of these putative NAC TFs could be regulated by ethylene, first a genome wide search for NAC domain TFs was performed, then expression profiles of selected candidates were examined by qPCR in fruit treated with ethylene at two stages of ripening (Figure 5, Supplementary Table 4). A total of 124 putative NAC domain encoding genes were identified in the oil palm genome, 33 of which had total read counts higher than 50 in the ripening mesocarp (Supplementary Table 4). A selection was made that consisted of the top five mesocarp expressed genes in addition to five additional genes with either down regulated or up regulated expression profiles in the ripening mesocarp. Gene specific primer sets amplified 10 non-redundant NAC sequences and confirmed their expression in the mesocarp (Supplementary Table 5). As with the studies on *EgACO* and *EgPG4*, ripe fruit at two stages (150 and 180 DAP) were treated with ethylene and the NAC transcripts were quantified. All the transcripts examined either increased or decreased at one time point in 150 or 180 DAP fruit. Of the 10 NAC transcripts examined, 7 NAC transcripts (*EgNAC2, 3, 5, 6, 7, 8*, and *13*) were significantly induced at one time point in either 150 or 180 DAP fruit. Notable, six of these (*EgNAC2, 3, 5, 6, 7*, and *8*) were induced after 3 h exposure to ethylene in the 180 DAP fruit, while two of those (*EgNAC5* and *6*) were also induced at 3 h in 150 DAP fruit. In addition, the *EgNAC5, 6, 7*, and *8* increased in abundance after 9 h of ethylene exposure in both 150 and 180 DAP fruit. *EgNAC6* and *7* were the most highly expressed transcripts expressed in response to ethylene in the mesocarp at both stages of ripening (150 and 180 DAP).

To provide insight into the diversification of NAC domain TFs, and to determine whether there is a relation between NAC domain structure and ethylene response, a phylogenetic analysis of the oil palm NAC domain sequences along with those of *Arabidopsis* and other selected NAC TFs with was performed (Supplementary Figure 2). Strong branch support values provided evidence for at least two major subclades of NAC domain sequences that resolve into a number of subgroups previously identified (Jensen et al., 2010). The oil palm mesocarp NAC domain sequences are distributed throughout the two major subclades, while six are found within the subgroups III-2 and III-3 (Supplementary Figure 2). NAC1, 2, and 3 group closely in subgroup III-3 and are also less abundant in the 150 DAP mesocarp after 3–6 h treatments with ethylene (Figure 5). In contrast, two of the most highly ethylene inducible NAC transcripts (*EgNAC6 and 7*) encode proteins found within subgroup III-2, along with the tomato NAC-NOR domain (Figures 5; Supplementary Figure 2). Among the other highly inducible transcripts; EgNAC5 separates in a less resolved portion of the cladogram, while EgNAC8 separates within subgroup II-3. It appears that an expansion of oil palm NAC domains occurred within the subgroups III-2 and III-3, where six sequences of the current study are found. In particular, two highly ethylene inducible genes, *EgNAC6* and EgNAC7, appear to be paralogous and encode NAC domain proteins the most homologous to the tomato NAC-NOR.

## Discussion

### The transition from ethylene production system 1 to 2 occurs concomitantly with a transcriptional activation of *EgACO*

The transition from basal auto inhibited ethylene production (system 1) to the burst of autocatalytic ethylene production (system 2) represents a central regulatory process that coordinates the expression of ripening related genes involved in pigmentation, aroma, carbohydrate, and cell wall metabolism (Seymour et al., 2013a; Liu et al., 2015). In tomato, the transition from system 1 to 2 is controlled both by developmental ethylene-independent and ethylene dependent factors and involves the transcriptional activation of specific ACS and ACO genes by the ripening related TFs Ripening-Inhibitor (RIN) and HB1 respectively (Nakatsuka et al., 1998; Barry and Giovannoni, 2007; Cara and Giovannoni, 2008; Lin et al., 2008; Yokotani et al., 2009; Klee and Giovannoni, 2011; Martel et al., 2011; Seymour et al., 2013a,b; Liu et al., 2015). The *EgACO* transcript is present in low amounts in the pollinated flowers (10 DAP), barely detectable or undetectable in at early stages of mesocarp development (30–100 DAP), and most highly expressed during the stages of fruit ripening (120–160 DAP), consistent with a role in both flower senescence and ripening similar to tomato (Blume and Grierson, 1997). However, the transcript profile differs from that observed during tomato ripening, in which the main ripening related *LeACO1* is expressed during both ethylene system 1 and system 2 stages of fruit development, with the highest expression during ripening and the autocatalytic system 2 (Holdsworth et al., 1987; Barry et al., 1996; Blume and Grierson, 1997; Nakatsuka et al., 1998; Jafari et al., 2013). Indeed, *LeACO1* is thought to participate in both system 1 and 2, and is not specific to ripening (Barry and Giovannoni, 2007; Cara and Giovannoni, 2008). Similarly in banana, the principle *MA-ACO1* transcript is also present in system 1 stage fruit, increases during the transition from system 1 to 2, and remains high at later stages of ripening (Liu et al., 1999). Our previous analysis revealed a burst of ethylene occurred in the oil palm fruit between 120 and 160 DAP, along with changes in transcript profiles related to ethylene biosynthesis and response between 100 and 160 DAP that reflect a transition from system 1 to system 2 ethylene production (Tranbarger et al., 2011). In contrast to tomato and banana, *EgACO* expression in the mesocarp is consistent with functions during the transition from system 1 to 2 (between 100 DAP and 140 DAP), and during ripening system 2 (between 140 and 160 DAP), but not during system 1 (100 DAP).

As in tomato, the increase in *EgACO* transcript during the transition from system 1 to 2 could be controlled either by ethylene independent or dependent regulation (Blume and Grierson, 1997). A comparison of *EgACO* and *EgPG4* transcript profiles during development and ripening and in response to ethylene suggests different modes of regulation in the mesocarp. *EgPG4* is barely detectable at the end of the transition from system 1 to system 2 ethylene production (approximated at 140 DAP), then increases dramatically at the autocatalytic ethylene stage (160 DAP), suggesting *EgPG4* expression may require a higher ethylene concentration threshold and/or does not respond to the same signals as *EgACO* during the transition from system 1 to 2. In contrast, expression of the principle tomato fruit TFPG increases more synchronistically with the increase in ethylene production (Dellapenna et al., 1986). In addition, there are at least two PGs expressed during banana ripening, one of which is the most highly expressed during ripening, and appears to be induced by ethylene (Asif and Nath, 2005; Asif et al., 2014). However, the ethylene treatments were for days and not hours as in the current study, so it is difficult to compare the results. In oil palm, the fact that *EgPG4* is consistently more highly induced by ethylene than *EgACO* at all treatment time points and stages examined suggests *EgPG4* expression is controlled differently by ethylene than *EgACO*, and argues against a higher ethylene threshold requirement for *EgPG4* regulation. The expression of *EgACO* during the transition from system 1 to 2 suggests developmental ethylene independent signals could induce *EgACO* during the transition, and not ethylene alone. One example of this in tomato is the LeHB-1 homeobox protein tomato that binds to the *LeACO1* promoter, while the inhibition of LeHB-1 mRNA results in reduced *LeACO1* mRNA (Lin et al., 2008). A search of the oil palm mesocarp transcriptome for sequences similar to LeHB-1 resulted in sequences with very low e-values (1e–26 to 1e–29) and low read counts, and therefore no evidence for a similar module was observed. Overall, while gene expression of key genes during the transition from system 1 to 2 in the oil palm mesocarp shares some similarities to that of banana and tomato, the examples of *EgACO* and *EgPG4* provided here suggest differences.

### The mesocarp undergoes a transcriptional transition from cell wall synthesis to degradation during ripening

The cell wall is an extremely complex structure made up of interlaced cellulose and hemicellulose polysaccharide polymers with structural glycoproteins embedded in a pectin matrix including HG, rhamnogalacturonan I and rhamnogalacturonan II. Unsurprisingly, many cell wall modifying proteins appear to function during ripening related cell wall disassembly and fruit softening. Attempts to silence single genes encoding only one type of modification have had little success to stop softening in the tomato fruit (Brummell, 2006; Seymour et al., 2013a). The identity of cell wall related transcripts identified in the current study that are differentially expressed during mesocarp ripening suggest a transcriptional based transition from synthesis to hydrolysis of the major cell wall polymers occurs in the mesocarp during ripening. Importantly, a single PG transcript (*EgPG4*) accounts for a large percentage of the total transcripts identified, while *EgPG4* expressed at a later stage of ripening, and is also induced by ethylene, similar to TFPG *PG2* transcript of tomato (Dellapenna et al., 1987). In addition, a number of other transcripts encoding various cell wall modifying activities were observed to have similar profiles to *EgPG4*, including glucan endo-1,3-beta-glucosidase-like, lichenase-2-like, chitinase 2-like, xyloglucan endotransglucosylase/hydrolase protein 23, UDP-glucose 6-dehydrogenase 5-like proteins were identified (Figure 3). In contrast, there are also a number of cell wall related transcripts with expression profiles similar to the ethylene biosynthesis transcript *EgACO*, also induced by ethylene, including, beta-galactosidase-like, alpha-glucosidase, and expansin-A10-like. Indeed, while *EgACO* and *EgPG4* are both induced by ethylene and are expressed in a ripening dependent manner, their ethylene induction and ripening expression profiles are different. That *EgACO* and *EgPG4* share similar expression profiles with various cell wall modifying protein transcripts suggests ethylene may transcriptionally regulate cell wall activities differentially as proposed previously (Ireland et al., 2014). Previous work on cell wall related gene expression during oil palm mesocarp ripening monitored only very few genes (Teh et al., 2014). In contrast, the current work includes a more comprehensive analysis of 56 differentially expressed genes from at least 20 different cell wall related gene families. The results suggest a close relation between the transcriptional activation of genes encoding expansin, polygalacturonase, mannosidases, beta-galactosidase, and xyloglucan endotransglucosylase/hydrolase proteins in the ripening oil palm mesocarp, comparable to that observed during tomato fruit ripening (Seymour et al., 2013a). While there is less available data with monocot fruit species, expansin and xyloglucan endotransglucosylase/hydrolase protein genes were also highly expressed during banana ripening (Asif et al., 2014). Overall, these observations provide evidence for the conservation of a large number of cell wall related activities that may be regulated by ethylene and other fruit ripening specific regulatory factors in diverse lineages of both eudicots and monocots.

### Paralogous members of the NAC domain family of transcription factors are regulated in the mesocarp during ripening and induced by ethylene

The NAC gene family is a very large group that encodes plant specific TFs involved in many processes including secondary cell wall development, stress response, and leaf senescence (Puranik et al., 2012; Kim et al., 2016; Monniaux and Hay, 2016). In addition, NAC TFs are upstream of ethylene related transcriptional regulation during both eudicot and monocot fruit ripening, and the NAC-NOR is a master regulator of tomato fruit ripening (Giovannoni, 2004; Shan et al., 2012; Seymour et al., 2013a; Kou et al., 2016). From a previous study we observed a number of NAC domain family TF genes that were differentially expressed during oil palm fruit ripening suggesting these TFs may function in response to and/or during the ethylene burst (Tranbarger et al., 2011). In the current genome wide study, we identified at least 31 NAC domain transcripts expressed in the oil palm mesocarp during the stages when ethylene production increases. Experiments with ethylene treated fruit revealed at least four have enhanced expression in the presence of ethylene. Furthermore, two of these oil palm NAC TF genes, *EgNAC6* and *EgNAC7*, appear to encode paralogous proteins that are very similar to the tomato NAC-NOR, suggesting a conserved function in the oil palm ripening mesocarp related to ethylene. Interestingly, the ethylene induction profiles of all *EgNACs* resemble more closely the induction profiles of *EgACO* than *EgPG4*, which suggests similar modes of regulation between the *EgNACs* and *EgACO* in the mesocarp. In tomato and banana NAC domain TFs are expressed during ripening and in response to ethylene (Shan et al., 2012; Zhu et al., 2014; Kou et al., 2016). In tomato, NAC family members may regulate the transcription of *LeACS2, LeACS4*, and *LeACO1*, while in banana, NAC domain TFs may regulate ethylene signal transduction through interactions with the downstream component ethylene insensitive 3 (EIN3)-like protein, termed MaEIL5 and the biosynthesis of ethylene (Shan et al., 2012; Kou et al., 2016). In the oil palm mesocarp, the ripening related and ethylene induced expression of several NAC-domain family members, in particular two with the most similarity to the tomato NAC-NOR, suggest a conservation of function for NAC domain TFs during the ripening of diverse lineages of both monocot and eudicot fruit types.

## Author contributions

TT and FM devised and participated in all aspects of the study. TT, CJ, and ST coordinated the logistics for fieldwork experiments. TT, FM, PR, CJ, PS, and MP performed the ethylene experiments and collected samples for RNA isolation and histological studies. PR extracted total RNA, isolated polygalacturonase cDNAs, performed cloning, designed gene specific primers, and performed preliminary RT-PCR expression studies. KF participated in the identification of the ACO and NAC domain cDNAs. MP, KF, CJ, and FM performed the qPCR analysis. TT performed the phylogenetic analysis. SD performed the statistical analysis of the transcriptome data. JV, PR, and MC prepared samples for histological analysis and performed microscopic analyses. TT, FM, SD, and JV participated in writing the article. All authors read and approved the final submitted manuscript.

## Funding

Financial support for this project came from PHC Thailande projects 2007-2010 (codes 20621YD and 16589YK) to TT and ST, and from PalmElit SAS/IRD/CIRAD to FM and TT. PR was supported by a Fondation Agropolis RTRA doctoral grant. KF was supported by a doctoral scholarship grant from the French Embassy with cofounding from PalmElit SAS.

### Conflict of interest statement

The authors declare that the research was conducted in the absence of any commercial or financial relationships that could be construed as a potential conflict of interest.

## Supplementary material

Supplementary Figure 1 **Mesocarp cell characteristics change during development and after 9 h ethylene treatment. (A)** longitudinal sections of mesocarp cells toward the base of the fruit stained with toluidine blue (upper panels) and ruthenium red (lower panels). **(B)** Cell wall, middle lamella, and cell wall width of mesocarp cells during development and after 9 h ethylene treatment. The different lower case letters represent statistical significance; the error bars represent standard error; cs, cell separation; n, number of samples measured; dc, divided cell; nd, no data; IM, intercellular material; IS, intercellular space; ml, middle lamella; Scale bar, 10 μm. Figure is reorganized from data previously.

Supplementary Figure 2 **Phylogenetic analysis of NAC domains from oil palm and other species reveals clusters of ethylene response**. Green and red asterisks represent a significant increase or decrease respectively in transcript abundance in relation to ethylene treatments (for details see Figure 5). Branch support values are based on the aLRT statistical test (see Materials and Methods). Branches with branch values less than 50 were collapsed. ng, not grouped.

Supplementary Table 1 **Cell wall mined**.

Supplementary Table 2 **Cell wall**.

Supplementary Table 3 **Cell_wall_global**.

Supplementary Table 4 **NAC Illumina**.

Supplementary Table 5 **NAC primers**.
