# Supplementary figures and images for: Transcriptome Analysis of Cell Wall and NAC Domain Transcription Factor Genes during Elaeis guineensis Fruit Ripening: Evidence for Widespread Conservation within Monocot and Eudicot Lineages
Source: Front Plant Sci. 2017 Apr 25;8:603. doi: 10.3389/fpls.2017.00603 (PMC5404384; doi:10.3389/fpls.2017.00603)

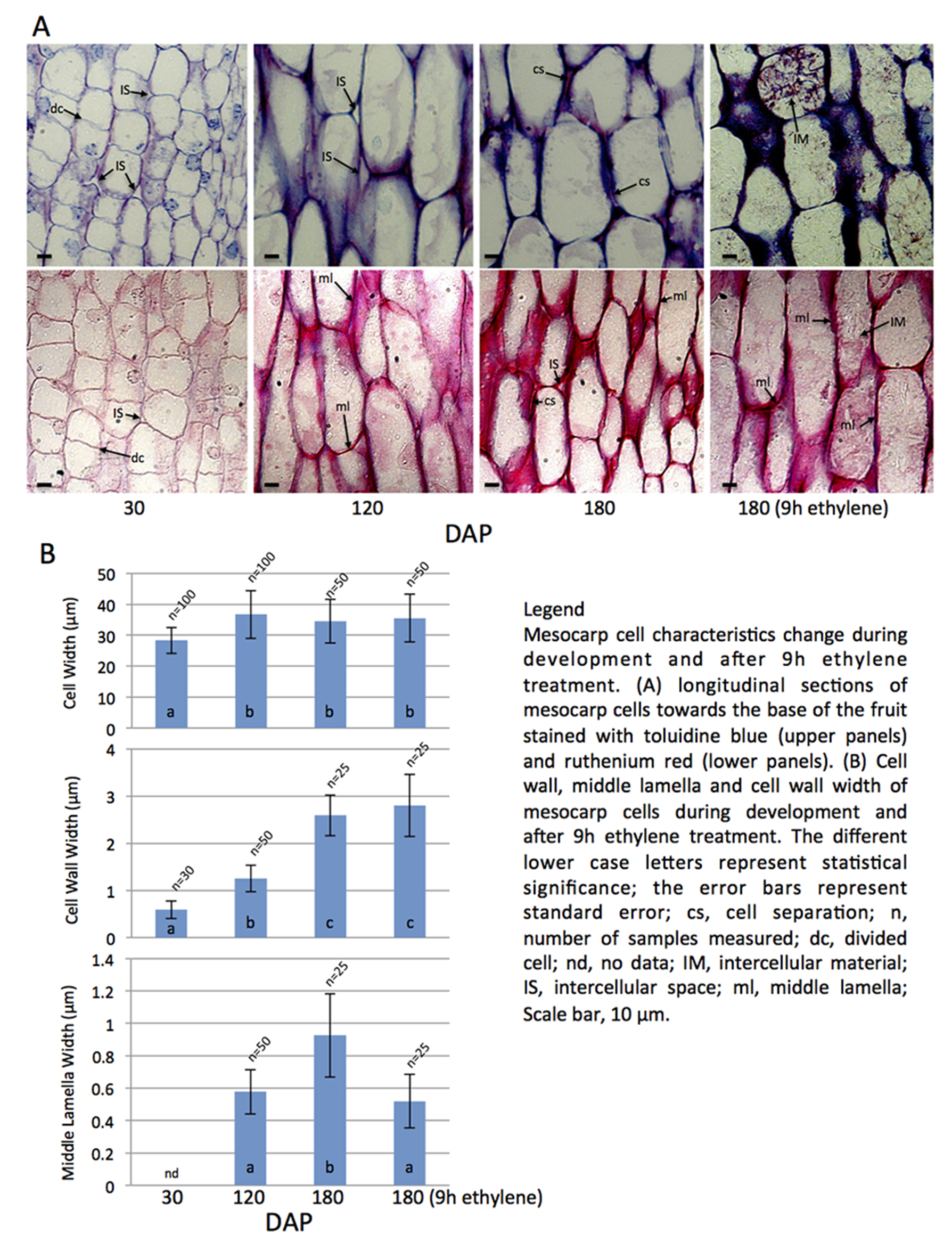

Supplement: Supplementary Figure 1 — Mesocarp cell characteristics change during development and after 9 h ethylene treatment. (A) longitudinal sections of mesocarp cells toward the base of the fruit stained with toluidine blue (upper panels) and ruthenium red (lower panels). (B) Cell wall, middle lamella, and cell wall width of mesocarp cells during development and after 9 h ethylene treatment. The different lower case letters represent statistical significance; the error bars represent standard error; cs, cell separation; n, number of samples measured; dc, divided cell; nd, no data; IM, intercellular material; IS, intercellular space; ml, middle lamella; Scale bar, 10 μm. Figure is reorganized from data previously. [file Image1.TIFF]

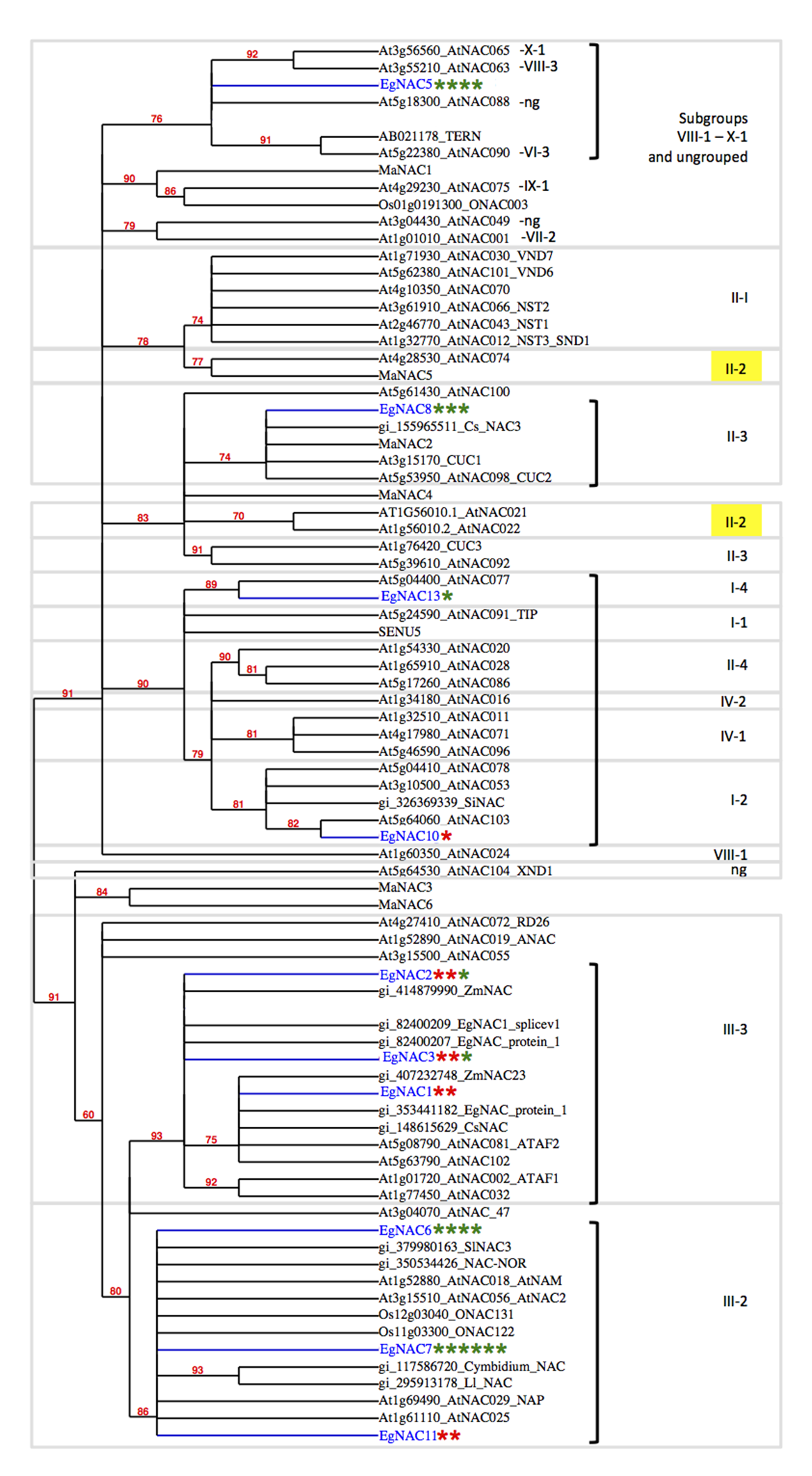

Supplement: Supplementary Figure 2 — Phylogenetic analysis of NAC domains from oil palm and other species reveals clusters of ethylene response. Green and red asterisks represent a significant increase or decrease respectively in transcript abundance in relation to ethylene treatments (for details see Figure 5). Branch support values are based on the aLRT statistical test (see Materials and Methods). Branches with branch values less than 50 were collapsed. ng, not grouped. [file Image2.TIFF]
