# Supplementary material for: Transcriptome Analysis of Cell Wall and NAC Domain Transcription Factor Genes during Elaeis guineensis Fruit Ripening: Evidence for Widespread Conservation within Monocot and Eudicot Lineages
Source: Front Plant Sci. 2017 Apr 25;8:603. doi: 10.3389/fpls.2017.00603 (PMC5404384; doi:10.3389/fpls.2017.00603)
Supplement: Supplementary Table 5 — NAC primers. [file Table5.DOCX]

**Supplementary Table 5 List of primers for the expression analysis of oil palm NAC domain genes by qPCR.**

| **Name of genes** | **Sequence of primers** |
| --- | --- |
| EgNAC1 | **Forward CTGAGCGACAGAAGGAGGAT**  **Reverse CGGCTTCAGATACGGAGAAC** |
| EgNAC2 | **Forward AAGCCAAAGGGTATGTCGTG**  **Reverse ATGATGCCTCCTTTTGTTGG** |
| EgNAC3 | **Forward ACGGACCTGAATCTGGATGA**  **Reverse CAGCTGGGATTCGTAGCTTT** |
| EgNAC5 | **Forward AAGACTGTTGGCAGGATTGC**  **Reverse TCTTGTTAGCGGTTGGTTCC** |
| EgNAC6 | **Forward CAGTACAATTTGTTAGGCCATCC**  **Reverse AAGCCTATACAAAATAAGGGCAATA** |
| EgNAC7 | **Forward CCCAAACTGCAACTTGCTAAA**  **Reverse GGCCCATCTCACTCACAAAT** |
| EgNAC8 | **Forward GAACACCGAGATTTCCTCCA**  **Reverse CAAGGCCAATTCTCTTCTGC** |
| EgNAC10 | **Forward TGATCAGCCCATCTTGTTTG**  **Reverse CCCTGAGCACACAAATCCTC** |
| EgNAC11 | **Forward AAAGGTCGGGGTGAAGAAAG**  **Reverse GTCAGCCTGCCGTTATTGTT** |
| EgNAC13 | **Forward TGGGAACTGCTGCTCTAATTTTAT**  **Reverse ACAAGTCGAGGACCTATAATCCAG** |
|  |  |
